# Delineating the Benefits of Arts Education for Children’s Socioemotional Development

**DOI:** 10.3389/fpsyg.2021.624712

**Published:** 2021-05-13

**Authors:** Steven J. Holochwost, Thalia R. Goldstein, Dennie Palmer Wolf

**Affiliations:** ^1^WolfBrown, Cambridge, MA, United States; ^2^Department of Psychology, Lehman College, City University of New York, Bronx, NY, United States; ^3^Department of Psychology, George Mason University, Fairfax, VA, United States

**Keywords:** arts education, theater education, socioemotional development, social awareness, relationship skills

## Abstract

In this paper, we argue that in order for the study of arts education to continue to advance, we must delineate the effects of particular forms of arts education, offered in certain contexts, on specific domains of children’s socioemotional development. We explain why formulating precise hypotheses about the effects of arts education on children’s socioemotional development requires a differentiated definition of each arts education program or activity in question, as well as a consideration of both the immediate and broader contexts in which that program or activity occurs. We then offer the New Victory Theater’s Schools with Performing Arts Reach Kids (SPARK) program as an illustrative example of how these considerations allow for the refinement of hypotheses about the impact of arts education on children’s socioemotional development.

## Introduction

Although research on the psychological benefits of arts education is expanding rapidly, problems remain in the ways in which such research is presented, publicized, and used to inform educational programs and policy. Chief among these is a tendency for discussions to focus on the benefits of “arts education,” as though all arts education were a monolithic activity with a singular pathway to uniform benefits. Here, we argue that our field must move beyond such broad claims about the impact of “arts education” to delineate the effects of particular forms of arts education, offered in certain contexts, on specific domains of children’s socioemotional development, a broad construct that encompasses identity formation, self-regulation, and interpersonal skills (Collaborative for Academic, Social, and Emotional Learning, 2021) and one that research increasingly suggests is fostered by many arts education experiences (see, for example, Farrington et al., 2019).

This specificity is essential for three reasons. First, the field has by now progressed to a point that merely demonstrating an association between some broad characterization of arts education (e.g., “theater education”) and some domain(s) of children’s socioemotional development (e.g., empathy) is unlikely to constitute a meaningful advance in our understanding of the relation between arts education and child development. In order to continue to build a scientific understanding of the potential role of arts education in children’s socioemotional development, we must formulate and test more precise hypotheses that link a particular form of arts education offered in a given context to a specific domain of that development. Only when all three of these terms – educational experience, context, and domain of socioemotional development – are adequately defined is it possible for researchers to reconcile the results of different studies and make informed hypotheses about whether the arts education experience that they are studying will yield a similar pattern of findings.

Second, if arts educators want to contribute to burgeoning efforts to foster children’s socioemotional development, they must design and implement programs that can accomplish this goal. This is far more likely when programs are intentionally designed around a plausible theory of change that links program activities to specific domains of children’s socioemotional development, and that provides guidelines for implementing a program with fidelity across different participants, sites, and contexts. The alternative – offering an ill-defined program and hoping for some unspecified socioemotional benefit to accrue – is unlikely to achieve results.

Third, just as a program is more likely to achieve its aims when built around a plausible theory of change, so too are initiatives or efforts comprised of many organizations working in concert. Given that arts education initiatives are often supported with public funds, educators and policymakers must be convinced of the initiatives’ potential prior to implementation and continued efficacy thereafter in order to provide support. Delineating the specific benefits of arts education initiatives to children’s socioemotional development aligns the expectations for these initiatives to the activities they offer and ensures that claims for these initiatives do not outpace the evidence for their likely effects.

These reasons could just as easily be cited to support an argument for a more thoughtful approach to understanding the benefits of the arts for children’s *cognitive* development, rather than their socioemotional development. Indeed, the boundary between cognitive and socioemotional development is often quite permeable: there is a cognitive component to most socioemotional skills and a socioemotional component to most cognitive abilities. Moreover, the effects of an arts education experience on a particular aspect of children’s socioemotional development (e.g., empathy) may be mediated by changes in children’s cognitive processes (e.g., theory of mind).

However, this paper focuses on arts education and children’s socioemotional development for two reasons. First, it is an area of burgeoning research interest, with an ever-increasing number of studies yielding findings that are now in need of conceptual organization. Second, it is also an area of emergent interest among educational practitioners and policymakers, and, as such, the socioemotional benefits of the arts have increasingly been cited in arguments that an education in the arts is an integral part of every child’s development (see, for example, the United Nations’ Convention on the Rights of the Child, Articles 28 and 29). That said, many, if not all, aspects of our argument would apply equally well to research that seeks to understand the effects of arts education experiences on children’s cognitive development, and we would encourage researchers whose work focuses on arts education and cognitive development to employ an approach similar to that which we outline here.

The remainder of this paper is divided into two sections. The first section reviews three aspects of any arts education activity or program that must be considered to effectively delineate its benefits on children’s socioemotional development: (1) a sufficiently differentiated definition of the arts education activity; (2) the immediate context in which that activity occurs; and (3) the broader ecological or environmental context in which it occurs as well. The second section proposes how researchers might frame hypotheses about the likely effects of a specific arts education intervention on children’s socioemotional development using the example of the New Victory Theater’s Schools with Performing Arts Reach Kids (SPARK) program – a theater program offered to students in the upper elementary grades.

## Delineating the Benefits of Arts Education

### Toward a Differentiated Definition of Arts Education

The first step in formulating precise hypotheses about the socioemotional benefits of arts education is to develop a differentiated definition of arts education activities and programs. While it may seem obvious that participation in a Ballet class is different than participation in a jazz music ensemble, as mentioned above, “arts education” is often treated as a monolith. Yet, there are not only distinctions both between art forms, but also within individual art forms as well (e.g., genre or tradition). It is an open question as to *whether* these differences cause variation in outcomes, and *which* elements of an arts class drive causal changes. Any researcher must decide at the outset, for example, whether they are interested in the holistic effects of a theater class, with its curriculum decided by experts in theater and its activities shaped over many years (e.g., Goldstein et al., 2017), or whether they would rather specify and isolate effects of an acting class *via* well-matched control groups and strictly-specified activities. Regardless, when discussing and reporting any research, details matter, as they define the specific opportunities for socioemotional development different arts education experiences and programs afford children (Gibson, 1979; Jenkins, 2008). These include, for example, whether the arts activities were experienced as audience or performer, and whether the arts practice was informed by classical forms, modern techniques, or post-modern experimental methods.

Two reasons such differentiation is not regularly undertaken in research reporting is because of the sheer number of ways in which arts activities can be categorized, and a lack of knowledge of which of these categorizations matters for children’s development. To begin, there is the **domain** of an arts education experience: (1) visual arts, including painting, drawing, sculpture, and collage; (2) dance, including ballet, jazz, tap, hip-hop, modern, and choreography; (3) theater, including improvisation, classical, modern, experimental, and musical theater; and (4) music, including orchestral, pop, jazz, band, and improvisation, performed either instrumentally or vocally, as well as media. This, of course, is a short and introductory list of possibilities and subgenres. Some scholastic curricula also include digital media or culinary skills in the arts, or separate out creative writing such as poetry, drama, or fiction into the arts, while others include creative writing genres in drama or English language classes.

It is important when thinking about the contextual effects of different arts domains on outcomes to keep in mind that art forms are often combined in practice, professional presentation, and occasionally in the classroom. Poems are set to music. Staging an opera requires music, dance, acting, and make up, costumes, and designed sets. Thus, while arts education experiences can be categorized in ways that reflect the disciplinary boundaries of the arts themselves, the boundaries between those experiences may be more or less permeable than those encountered in the arts the arts themselves. Moreover, elements of the arts may be integrated into educational experiences that are primarily intended to convey knowledge about subjects outside of the arts, such as when the visual arts are used to teach geometry or when theater is used to enliven history lessons (Hardiman et al., 2014; The Kennedy Center, 2020). While complex, studying how teachers separate and combine artistic domains will best allow researchers to approximate both the intricacies of real-world practice and the rigor necessary to form conclusions about how the arts affect socioemotional development.

Each domain of the arts has non-mutually exclusive **characteristics** which can specify effects. Music, theater, and dance are generally interpretative and collaborative. Musicians, dancers, and actors can perform solo or work in ensembles of many sizes, learning and interpreting a composer’s, choreographer’s, or playwright’s work. Visual artists, in contrast, tend to work more by themselves, generating material. However, visual artists can work in collectives, and music, dance, and theater all have the possibility of generating and/or improvising work as part of study. In fact, most theater classes begin with an improvisational warm up, and use the generation of text and behavior throughout rehearsal processes. Music and dance both rely on rhythm; theater and the visual arts contain figural and representative elements. Within each domain and genre, an additional element to consider is the time period or form on which the class is focused. Any class in these arts domains could focus on Western or Eastern classical works, the modern artistic revolutions of the 19th and 20th centuries, or current experimental work. Like any area of study that continues to be informed by its own history, arts classes’ foci in time affect the type of work the student will engage in, their freedom of form and interpretation, and the rules they “should” follow. Whether these differences lead to distinct outcomes is unstudied at this point.

Similarly, there may be fundamental differences when a student actively participates in the creation of art, theater, dance, or music, compared to when they are simply in the audience or observing. While there is some evidence to suggest that both watching (Greene et al., 2018) and participating (Goldstein and Winner, 2012) in theater in middle childhood positively affects empathy, more studies are needed to replicate both effects, and this type of convergence may not hold for other art forms. Painting or walking through a museum, playing a violin or sitting in a concert hall, hours of physical practice or watching a Ballet are such significantly different behavioral and psychological activities, it would be very surprising if they caused the same effects.

One starting point for conceptualizing the real implications for social and emotional learning across and within art domains is by investigating the habits of mind fostered and supported by each. Habits of mind are cognitive patterns – domain general ways of thinking about problems, framing the world, and guiding behaviors (Perkins et al., 1993). Intensive studies on habits of mind are well-established in the visual arts (Hetland et al., 2015), with similar studies recently conducted in music (Hogan and Winner, 2019) and theater (Goldstein and Young, 2019). The similarities among art forms, such as their aesthetic and expressive components, have led some theorists to work toward a unification of the psychological components of art forms (Brown, 2018), but practitioners may or may not agree. To this point, both visual arts and music have been found to employ the habits of mind of persistence (i.e., keep going at practice and working through a problem); imagination (of what changes in a musical performance or visual stimuli may look like); and expression of ideas and meaning (Hetland et al., 2007, 2015; Hogan and Winner, 2019). But music may focus on building and creating ensemble while visual arts engage the use of careful observation and perception.

Finally, the “same” artistic activity can occur in many forms (Greeno, 2006). To take an example from theater education, a child may study his character, memorize lines, rehearse scenes, informally perform for peers, or perform in a full-fledged production before an audience. Each of these activities has different experiential elements and immediate contexts, and, as such, may inculcate different states of arousal and incur different consequences. Thus, if researchers seek to build the evidence base for incorporating theater education into school curricula and youth programming, it is vital to understand which activities in which contexts have a measurable impact on what domains of socioemotional development among which children (Holochwost et al., 2018).

### The Role of Context

#### The Immediate Context

Defining an arts education activity or program by differentiating it in terms of its domain and characteristics is an essential first step toward formulating hypotheses about that activity or program’s socioemotional benefits. The next step is to consider the immediate context in which that activity or program occurs. The purpose of this is to provide a deeper understanding of where, for whom, by whom, and how a specific arts education experience was offered. For example, a performing arts residency program could play out quite differently in an arts magnet elementary school and an elementary school that lost its arts programs a decade ago. Similarly, the impact of a performing arts program might be markedly different if classroom teachers are viewed chiefly as behavior managers and facilitators or if they are active participants in professional development sessions designed to transfer performing arts strategies to their daily instruction. Taken together, information on the immediate context helps to define the environment/ecology in which a program occurs, who is an active participant, and how the program was implemented.

One essential parameter of the immediate context of an arts education program or activity is the **specific institutional setting** in which that activity occurs. A good deal of arts education occurs in schools, but arts education also takes place in many other settings, from community arts organizations to cultural providers to children’s homes. Each of these settings has a particular **arts learning profile**, a configuration of characteristics that defines that setting as an immediate context for arts learning. For programs that occur in schools, elements of this profile include the adequacy of the physical space made available for the program, the level of support offered to the programs by classroom teachers and administrators, the history and prominence of arts education at the school, and whether arts education is part of the curriculum for all students or whether it is made available only to students who meet certain academic or behavioral standards. Merely knowing that a student participated in a program of music education at their school is insufficient; the arts learning profile of a school with no dedicated practice or performance space and a single, itinerant music teacher could not be more different than that of a well-resourced arts magnet school.

Another key parameter is whether there is someone who guides or directs the arts education program or activity. While some arts education experiences may be self-directed, even an apparently independent learning experience such as roaming a museum exhibit is guided by curatorial decisions and placard texts. However, many arts education experiences feature a more prominent guide in the form of a teacher or teaching artist, and in these cases that **teacher’s characteristics** become important aspects of the immediate context of arts education (Diamond, 2015). These may include the teacher’s personal characteristics (e.g., gender and ethnic identities), training (both as an artist and an educator, including access to and use of professional development), their experience (again, as an artist and educator in general, but also as arts educator in comparable settings), and their role in the institutional setting (e.g., full-time faculty, itinerant faculty, or guest artist).

Finally, there are the **characteristics of the program** or activity as delivered in practice. As Diamond and Ling observed, “the ‘same’ program or intervention can be administered differently by different individuals,” and the benefits of any program to children’s socioemotional development will be determined by children’s experience in that program as it is delivered to them (Diamond and Ling, 2020, p. 366). The overall quality of that experience will be defined largely by its process quality (Zaslow et al., 2010), or the patterns of interaction between teacher or teaching artist and child.

Studies of early education have consistently revealed that teachers’ sensitivity when interacting with children is the principal determinant of whether children derive benefits from early education programs (Burchinal et al., 2000; Melhuish et al., 2015). Similarly, sports and athletic enrichment programs have been found to be most beneficial for children when coaches refrain from negative behaviors in their interactions with children (such as embarrassing children) and instead exhibit sensitive behaviors such as offering praise and encouragement, and emphasizing teamwork and enjoyment (Smoll et al., 1993; Smith and Smoll, 1997). Indeed, the benefits children derive from any arts education program will be contingent upon their engagement in that program, and engagement is based, in part, on enjoyment (Ericsson et al., 2009).

Even a very high-quality program must exceed some minimal threshold of dosage in order for it to yield benefits to children’s socioemotional development. Dosage may be defined by the frequency and duration of the program or activity across two time scales (minutes per experience and time between the first experience and the last). The dosage of arts education experiences ranges widely from a single field trip to see a performance to daily instruction that spans the course of childhood. All else being equal, higher dosage of an arts education program or experience would be expected to predict greater benefits to socioemotional development, but only when that program or experience clears some minimal threshold for dosage.

#### The Ecological or Environmental Context

As the prior section makes clear, arts education activities and programs are not untethered abstractions; they happen in a given institutional setting with a unique arts learning profile, and are delivered according to a particular model (which may include the presence of a teacher) at a particular dosage. Moreover, the immediate context in which arts education activities and programs occur is nested within a broader ecological or environmental context.

The most important component of this broader context is the child or children who are being educated, without whom any arts education activity or program cannot occur. The characteristics or features internal to the student or students are, therefore, a key aspect of ecological or environment context in which arts education occurs. Theoretically, almost any child factor could influence the potential for an arts education activity or program to benefit a particular domain of children’s socioemotional development. But some of those factors have proven most likely to have an effect in the greatest number of instances, beginning with the factors that are internal to the student.

Among these factors, age or developmental stage may be the most important influencer across the widest array of situations, due to the trajectories of different domains of socioemotional development. These trajectories influence how sensitive or malleable these domains are when a child participates in a particular arts education program or experience. Consider the example of self-concept. Even very young children have a concept of themselves; however, among young children self-concept is very broad and general. As children age, self-concept becomes increasingly nuanced. By middle childhood, children reliably differentiate between their self-concept with respect to academics and their self-concept in athletics; by adolescence, they see themselves differently in the context of different academic subjects (Marsh and Ayotte, 2003; Marsh et al., 2018).

As a result, an arts education program designed to enhance academic self-concept among preschoolers could not reasonably be expected to achieve that aim, for the simple reason that children at this age do not have an academic self-concept to enhance. All else being equal, a program that targeted academic self-concept among adolescents would be more likely to succeed, if its design and implementation reflected not only the trajectory of self-concept across adolescence, but also the ways in which that trajectory, combined with the relative malleability or recalcitrance of different aspects of self-concept, rendered those aspects targeted the program more or less open to change. Across different areas, self-concept in adolescence generally follows a curvilinear trajectory, in which self-concept is more positive as children enter adolescence, becomes more negative as adolescence proceeds, and then recovers as it ends. Depending when during adolescence an arts education program occurred, it might be expected to have different effects, though the precise nature of those effects would depend on whether more positive or more negative self-concept would be expected to constrain or promote the program’s potential benefits.

Other particularly salient child factors include gender and racial/ethnic identity. Returning to the example of self-concept, a meta-analysis found that school-aged males exhibited slightly higher academic self-concept scores than females (Wilgenbusch and Merrell, 1999). However, this overall difference masked the fact that this difference held only for academic self-concept with respect to mathematics; where English language arts was concerned, females reported higher levels of self-concept (Stetsenko et al., 2000; Kurtz-Costes et al., 2008). Hence, expectations for arts education to benefit children’s self-concept may have to be conditioned on both gender *and* the specific area of academic self-concept (here, math vs. English) that a program sought to change.

Of course, while we may seek to isolate the influence of different child factors on socioemotional development in our research designs (e.g., by holding different factors constant), any number of idiographic perspectives (e.g., social identity theory) reveal that within any particular child, these factors occur together (Sellers et al., 1998). That is, a child is not merely an adolescent, but a female adolescent (and many other things besides). The intersection of these factors will jointly impinge upon the effects of any arts education program on that child’s socioemotional development. For example, the magnitude of the gender difference in academic self-concept is three times larger in middle childhood (in males’ favor) than it is in adolescence (Wilgenbusch and Merrell, 1999).

Moreover, the influence of these factors will in turn be affected by elements of the developmental ecology that are external to the child. Most salient among these is the child’s family. Many aspects of the family have been linked to children’s socioemotional development, from family structure (Lee and McLanahan, 2015; Bzostek and Berger, 2017) to patterns of interaction between parents and children (Bridgett et al., 2015). Other family factors that have received far less attention, such as whether there is an artist in the immediate or extended family, may be particularly salient influences on whether arts education benefits children’s socioemotional development.

One level removed from the family are the elements of the developmental ecology that comprise Bronfenbrenner’s exosystem: the child’s peer group, school, and neighborhood (Bronfenbrenner and Morris, 2006). Each of these elements may also influence the impact of arts education of children’s socioemotional development, either directly or by impinging upon levels of the ecology that are more proximal to the child. For example, a lower-income family may reside in a neighborhood comprised of families with various incomes, or they may reside in a neighborhood of concentrated disadvantage. While the social comparison factors (Festinger, 1954) that may accompany living in a mixed-income neighborhood should not be overlooked, growing up in an area of concentrated disadvantage exerts a direct and tangible effect on children’s socioemotional development, above and beyond the effects of familial socioeconomic status (Carpiano et al., 2009; May et al., 2018). However, concentrated disadvantage may also exert an indirect effect on the benefits of an arts education program through its effects on child-level factors, by, for example, limiting a child’s access to arts education and thereby restricting their prior experience in the arts.

**Figure 1:**
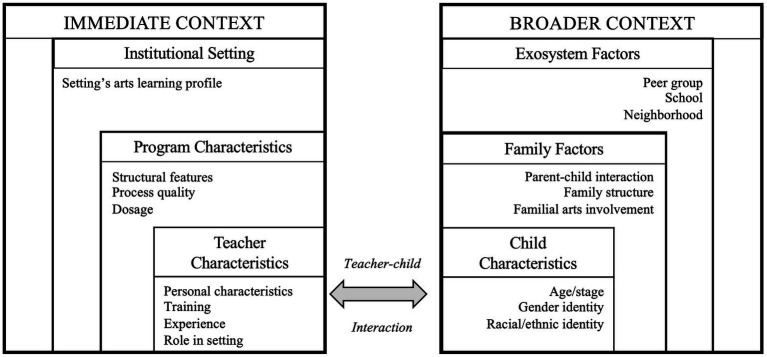
Graphical summary of immediate and broader contextual factors that may promote or constrain the benefits of arts education programs or experiences on children’s socioemotional development.

Figure 1 summarizes immediate and broader contextual factors discussed above that may promote or constrain the benefits of arts education programs or experiences for children’s socioemotional development. While this figure includes the factors discussed in the text, it is not intended to be an exhaustive list of all immediate and broader contextual factors that may impinge upon the benefits of arts education programs or experiences.

#### Formulating Hypotheses

Once a differentiated definition of a particular arts education experience or program has been established and the immediate and broader contexts in which that experience of program have been considered, the final step in formulating a hypothesis is to link that experience or program, as it occurs in those contexts, to a particular domain of children’s socioemotional development. Socioemotional development is typically defined quite broadly as “the process through which children and adults understand and manage emotions, set and achieve positive goals, feel and show empathy for others, establish and maintain positive relationships, and make responsible decisions” (Collaborative for Academic, Social, and Emotional Learning, 2021). This broad definition encompasses many distinguishable domains of development, including theory of mind, empathy, compassion, sympathy, emotion understanding, and self-regulation, to name a few. The challenge is to use theory and prior research, together with the definition of the arts education experience or program and knowledge about the contexts in which it occurs, to predict which domains of socioemotional development are most likely to be fostered by that experience or program. In the remainder of this paper, we use the example of the New Victory Theater’s SPARK Program to illustrate how this may be accomplished, and then provide a brief description of a research project designed to test the resulting hypotheses.

## An Illustrative Example: the New Victory Theater’s Spark Program

### A Differentiated Definition of the SPARK Program

The New Victory Theater’s SPARK program was designed to introduce the performance arts as a core element of the curriculum in schools without opportunities for arts education. The program was based around three performances that third-grade students attended over the course of a single school year, paired with 15, weekly, in-class workshops that were led by teaching artists (see immediate context below). These performances ranged from new plays and theatrical adaptations of existing stories (e.g., *Mr. Popper’s Penguins*) to less narrative, performances like circus arts productions and dance revues. Over the course of the year, students saw a balanced slate of productions, including one narrative drama, one circus arts production, and one performing arts revue. Regardless of genre, these productions featured many performers who were people of color playing major roles, and the content of the productions drew on the artistic and performance traditions of many cultures.

In-class residency sessions focused on teaching students information and vocabulary related to the productions they would see and the human lessons those performances embodied. For example, prior to seeing the performance of the circus arts production, *Mother Africa* students learned about the varied African origins of the performers and the long years of daily practice that they spent gaining the circus skills they performed in the show. The residencies also featured activities closely related to the performances students would see. For example, before going to see a circus arts performance, students learned how to perform simple tricks like scarf juggling and plate spinning. Throughout each workshop session, teaching artists taught many interpersonal skills, including subtle ones such as not laughing or teasing when a peer made a mistake or suffered a setback. Teaching artists would often recall a scene or situation from one of the narrative productions (e.g., *The Velveteen Rabbit*) and ask students to take the perspective of different characters, articulating those characters’ words and internal thoughts.

According to our differentiated definition, this was a theater program, but one that featured performance arts rather than theater alone. The predominant genre of works presented was modern, rather than historical or experimental, and many of the productions, as well as many of the activities featured in the workshops emphasized the human and ensemble nature of theater. Students’ participation in the program was multimodal: for the portion of the program in which they attended productions, students were members of the audience. However, students were also active participants during the residencies, contributing to generative theatrical and circus performance activities (rather than scripted activities).

### The Immediate and Environmental Contexts of SPARK

As described above, the immediate context for an arts education program or activity is comprised of the specific institutional setting with its unique arts learning profile, the presence of a teacher and their characteristics, and the dosage of the experience. In the case of SPARK, there were two institutional settings for the program: the theater, where students attended the three productions, and their classrooms, where the residencies took place. The New Victory Theater is a historic venue that was transformed into a children’s theater in the mid-1990s. It is located on Broadway, in the heart of New York City’s theater district. It is widely regarded as one of the premiere children’s theaters in the world, and is especially well-known for presenting complex works to young audiences. For many years, the theater has run a program that recruits young people of color as ushers in the theater, offering them paid employment while training them for careers in the performing arts.

The children who participated in the program were in one of four classrooms (all in a single grade) at an elementary school that had been identified by the New York City School District as underperforming. The school had no arts teachers on its faculty (either full or part-time) and was not being served by any other community-based arts education partners. However, school administrators were interested in using the arts as a strategy to engage students and improve the overall performance of the school.

The residency sessions were led by pairs of teaching artists (TAs) who were actors and performers working in New York City. In a number of cases, these TAs were working in other productions while the residencies were in progress; one TA was featured in the Broadway production of *The Lion King*; another was in the touring production of the Blue Man Group (as one of the Blue Men). In addition to the training they received when they were hired to be part of the SPARK program, TAs attended a series of professional development workshops presented by the New Victory Theater over the course of the program year.

The residency sessions were delivered during the school day, and generally during English language arts instruction. The same pair of TAs was assigned to the same classroom(s) throughout the school year. Classroom teachers and any paraprofessional remained in the classroom during the residency sessions, though their levels of involvement varied considerably. To foster a closer working relationship with the classroom teachers, the New Victory Theater hosted three teacher workshops over the course of the school year. The sessions lasted a full class session (approximately 40 min) and 15 sessions were held between October and May. These parameters of the immediate context are summarized in Table 1.

**Table 1:** Definitional and contextual parameters of an arts education experience.

*Parameter*	*Illustrative Example: New Victory Theater SPARK*
Differentiated Definition of Arts Education	
Domain	Theater
Genre/tradition/methods (e.g., classical, modern)	Mix of narrative and performance-art productions featuring the arts of many cultures
Combinations Multiple arts domains Integrated/combined with other subjects	Theater productions encompassing other disciplines (e.g., visual arts in set design), music, and dance
Characteristics Type of activity (solo/group) Mode of participation (passive/active)	Performance and residencies were group activities Children were audience members at performances and participants in the residency sessions
Immediate Context of a Program or Experience	
Specific institutional setting (e.g., school) Setting’s arts learning profile	The New Victory Theater: A historic theater in the City’s theater district One of the world’s premiere children’s theaters Ushers are young people of color Partner School: A low-performing, public elementary school No arts teachers on faculty No community-based arts education partners Supportive administration
Presence/characteristics of teachers/teaching artists: Personal characteristics Training (as artist and educator) Experience (as artist and educator) Role in institutional setting	Teaching artists (TAs) were actors and performers TAs received initial training and attended a series of professional development workshops at the New Victory Theater
Program Characteristics Structural features Process quality	Performances: Students were bused from their school to attend three performances at the New Victory Theater Residency: Sessions occurred during the school day in students’ classrooms (typically during English language arts) Sessions were always led by the same pair of TAs within each classroom
Dosage Frequency of instruction Duration of activity	Performances: Students attended three performances over the course of the academic year Residency: Fifteen 40-min sessions were held between October and May
Broader Context of a Program or Experience	
Child characteristics Age/developmental stage Gender identity Racial/ethnic identity	Children were in third grade 60% of students identified as female 76% of students identified as Hispanic, and 20% identified as Black
Exosystem factors	
Peer group School Neighborhood	90% of children attending the school received free or reduced-price lunch 57% of families with children in the school’s zip code were living in poverty

In the year that we worked with the program, the children who participating in it were in third grade. Students in fourth grade comprised a comparison group, and though these students attended one performance at the theater, they did not attend the other two, nor did they receive the in-class residency. Across these two groups, approximately 60% of the students were female; nearly all children who attended the school were of color (20% Black and 76% Hispanic). Over 90% of the students who attended the school received free or reduced-price lunch, and the school is located in an area of concentrated economic disadvantage (57% of families with children in the zip code in which the school is located were living in poverty).

### Hypotheses About the Program’s Effects

We anticipated that participating in the SPARK program would confer benefits across a number of domains of socioemotional development. For purposes of illustration, we will focus on how we formulated hypotheses about the potential for the program to foster students’ social awareness and relationship skills.

We defined social awareness and relationship skills as the abilities to take others’ perspective, to empathize with them, and to form positive relationships with their peers. On the basis of prior research, we hypothesized that participating in SPARK would be associated with an enhanced capacity to take others’ perspectives (Goldstein et al., 2013; Greene et al., 2018), higher levels of empathy (Goldstein and Winner, 2012), and more positive peer relations (DICE Consortium, 2010). Given that previous research has demonstrated the potential for attending a single theatrical performance to improve aspects of children’s perspective-taking abilities (Greene et al., 2018), we acknowledged that students assigned to the *comparison* group might exhibit improvements over baseline in this domain. However, we anticipated that the opportunity of treatment group students to attend multiple productions *and* participate in the residencies would lead to still greater gains.

We then refined this hypothesis in light of the differentiated definition of the SPARK program and both the immediate and broader contexts of the program. We anticipated that three specific aspects of the program might amplify its capacity to foster students’ social awareness and relationship skills. First, the productions students attended introduced students to the arts of different cultures and the capacity of human beings to imagine new possibilities. Second, the residencies explored the lives of both the performers and the characters included in the narrative productions. Third and finally, the residencies required that all students engage collaboratively in unfamiliar activities (e.g., scarf juggling) in front of their peers. We anticipated that by making each student vulnerable, the likelihood that each student would feel empathy for their peers when it was their turn to be vulnerable would be increased, while having students work together to accomplish these activities (and thereby mitigate their vulnerability) increased the chances that they would form supportive relationships with one another.

As for the immediate context, we expected that the arts learning profiles of the two settings in which the program occurred – the New Victory Theater and the students’ school – would work in tandem to further enhance the potential for the program to foster students’ social awareness and relationship skills. For nearly all students who participated in the program, attending the New Victory Theater was the first time they had traveled to New York City’s theater district, and, as such, represented an opportunity to increase their social awareness by seeing people doing things they had never seen a person do before (e.g., ride a unicycle, do a backflip, or deliver lines onstage). While this may be an eye-opening experience for any student, for a student from a school with no arts faculty and no other partnership programs, it may be revelatory.

In a similar vein, we anticipated that increases in students’ social awareness might be rendered more likely due to the characteristics of their teaching artists. Throughout the program, students displayed a keen interest in understanding how performers came to be able to do the amazing things they did during the shows students saw. When given the opportunity after each show to talk to the performers, students would ask them, but this topic would also come up once students discovered the TAs were talented performers in their own right. The delivery model for the program, in which TAs worked with the same classroom of students over the course of the year, allowed this initial curiosity to develop into an increased understanding of the TAs’ training and background on the part of the students, as well as the students’ interests and aspirations on the part of the TAs. Other aspects of the delivery model led us to expect that students would form positive relationships with each other. One of these was the fact that students attended performances as a classroom, providing them with a common touchstone of a special, shared experience. Another was that the residency occurred in students’ classrooms, allowing for the possibility that positive relationships formed in the context of the residency could carry-over to the broader context of the classroom when the residency was not in session.

Finally, there is the broader context, beginning with the characteristics of the child. The children in SPARK were in third grade at the time of their participation in the program, an age when social awareness and relationship skills are undergoing rapid consolidation (Collins, 1984). The fact that SPARK coincided with a sensitive period for the development of these skills raised the likelihood that the program would improve them. In our estimation, so too did two aspects of the environmental context. First, there was the fact that children participating in the program were almost entirely children of color who are, therefore, more likely to experience the types of racism and exclusion that can erode relationship skills (Pachter et al., 2010). Second, the children were disproportionately likely to be from families in poverty, another factor that can impede the development of relationship skills (Moilanen et al., 2010). We reasoned that the opportunity to participate in the SPARK program might mitigate the effects of racism and poverty on these skills, and that the magnitude of this effect may be larger, given the participants’ backgrounds of relative disadvantage (Catterall, 2012; Greene et al., 2013).

To test these hypotheses, we collected data from two groups of students: third-grade students who attended the productions at the New Victory Theater and participated in the residencies (designated as the treatment group) and their fourth-grade peers at the same school, who only attended the productions and were, therefore, designated as the comparison group. Prior to and following the program, students in the treatment group completed a set of measures designed to yield both quantitative and qualitative data; students in the comparison group completed the same measures according to the same schedule. In general, measures that yielded quantitative data were taken from existing measures (e.g., the empathy subscale from the Social Skills Rating Scales, or SSIS; Gresham and Elliott, 2008). However, we also designed a set of complementary measures that could yield richer information about the impacts of the program on children’s social awareness and relationship skills. For example, students completed an ecogram in which they were asked to imagine that they were forming their own theater company, and to assign classmates to the roles of actors, playwrights, directors, and designers. A structured sub-sample of students also completed a task in which they narrated a short, silent film that portrayed a character trying to escape from a mysteriously and invisibly locked park. Researchers instructed students to explain not only what was happening in the film, but what the character was thinking, feeling, and planning. The resulting stories were coded for information about the character’s internal states and life circumstances beyond what was shown in the film.

At this point, our data collection has concluded, but our analyses are ongoing. Regardless of the specific nature of the results ultimately yielded by these analyses, our ability to interpret those results will be enhanced by having formulated hypotheses that account for the differentiated definition of the arts education activity children experienced, and both the immediate and broader contexts in which that activity occurred. While all researchers prefer positive findings – in part because they are easier to publish – the field of arts education research is advanced more rapidly by studies with precisely-articulated hypotheses that yield null findings than studies featuring positive findings that are poorly motivated and contextualized and, therefore, difficult to interpret.

## Conclusion

As this example illustrates, using a differentiated definition of an arts education program and considering its immediate and broader contexts to specify the benefits of that program on children’s socioemotional development allows us to formulate more precise hypotheses about not only *what* benefits those programs may confer, but *how* those benefits may be conferred. This understanding is a pre-requisite for the intentional design of arts experiences designed to yield a particular benefit and for understanding how definitional and contextual factors make the realization of that benefit more or less likely. Just as important, this understanding is a hallmark of a maturing science, one that is able to progress beyond the observation of a phenomenon – such as the association between arts education and child development – to offering an explanation of that phenomenon.

As our example suggests, the promotion of socioemotional development through arts education may be an equifinal phenomenon, one in which many pathways lead to the same end. However, that does not lessen the value of understanding each of those pathways, as each may be the most efficient route to a particular socioemotional end for a particular population of children. At present, many of those paths are uncharted; for example, as a field we know very little about how the alignment of the cultures featured in performances and the cultures of origin for the children attending those performances might impact the likelihood of developmental in a particular socioemotional domain, just as we know little about the importance of students of color seeing performances by people who are also of color, or the marginal benefit of increased dosage for a particular domain of socioemotional development. However, by formulating precise hypotheses about the effects of arts education on children’s socioemotional development, we increase our chances of answering them in the fullness of time.

## Data Availability Statement

The original contributions presented in the study are included in the article/supplementary material, further inquiries can be directed to the corresponding author.

## Author Contributions

SH, TG, and DW conceptualized this manuscript. SH prepared the initial draft of the manuscript, to which all authors subsequently made contributions. All authors contributed to the article and approved the submitted version.

### Conflict of Interest

SH and DW were employed by company WolfBrown.

The remaining authors declare that the research was conducted in the absence of any commercial or financial relationships that could be construed as a potential conflict of interest.
